# Reconstruction of putative DNA virus from endogenous rice tungro bacilliform virus-like sequences in the rice genome: implications for integration and evolution

**DOI:** 10.1186/1471-2164-5-80

**Published:** 2004-10-18

**Authors:** Motoyuki Kunii, Masanori Kanda, Hironori Nagano, Ichiro Uyeda, Yuji Kishima, Yoshio Sano

**Affiliations:** 1 Laboratory of Plant Breeding, Graduate School of Agriculture, Hokkaido University, Sapporo 060-8589, Japan; 2 Laboratory of Plant Pathology, Graduate School of Agriculture, Hokkaido University, Sapporo 060-8589, Japan

## Abstract

**Background:**

Plant genomes contain various kinds of repetitive sequences such as transposable elements, microsatellites, tandem repeats and virus-like sequences. Most of them, with the exception of virus-like sequences, do not allow us to trace their origins nor to follow the process of their integration into the host genome. Recent discoveries of virus-like sequences in plant genomes led us to set the objective of elucidating the origin of the repetitive sequences. Endogenous rice tungro bacilliform virus (RTBV)-like sequences (ERTBVs) have been found throughout the rice genome. Here, we reconstructed putative virus structures from RTBV-like sequences in the rice genome and characterized to understand evolutionary implication, integration manner and involvements of endogenous virus segments in the corresponding disease response.

**Results:**

We have collected ERTBVs from the rice genomes. They contain rearranged structures and no intact ORFs. The identified ERTBV segments were shown to be phylogenetically divided into three clusters. For each phylogenetic cluster, we were able to make a consensus alignment for a circular virus-like structure carrying two complete ORFs. Comparisons of DNA and amino acid sequences suggested the closely relationship between ERTBV and RTBV. The *Oryza *AA-genome species vary in the ERTBV copy number. The species carrying low-copy-number of ERTBV segments have been reported to be extremely susceptible to RTBV. The DNA methylation state of the ERTBV sequences was correlated with their copy number in the genome.

**Conclusions:**

These ERTBV segments are unlikely to have functional potential as a virus. However, these sequences facilitate to establish putative virus that provided information underlying virus integration and evolutionary relationship with existing virus. Comparison of ERTBV among the *Oryza *AA-genome species allowed us to speculate a possible role of endogenous virus segments against its related disease.

## Background

The virus-like sequences that have been found in plant genomes are divided into two groups of plant viruses, single-stranded DNA geminivirus and double-stranded DNA pararetroviruses. The geminivirus segments, including the viral replication origin and the adjacent *AL1 *gene, have been found in the genomes of tobacco and its related species [1,2]. Pararetrovirus-like sequences have been reported in the petunia [3,4] banana [5-7] and tobacco genomes [8-10]. Compared to the intact virus sequences, most of the endogenous virus-like sequences were rearranged in the host genomes. Their rearranged structures suggested that illegitimate recombination may have occurred when putative virus progenitors integrated [11]. The endogenous viruses for banana streak virus (BSV) [6], tobacco vein-clearing virus (TVCV) [9] and petunia vein clearing-virus (PVCV) [4] could be activated as episomal viruses under certain conditions in the host plant, and appeared to have pathogenic potential. The integrations of these viruses were shown to have been relatively recent events and the copy numbers of the endogenous virus sequences were found to be very low. On the other hand, for tobacco endogenous pararetroviruses (TEPRVs), it was estimated that there are about 1000 segments in the tobacco genome [8], but the intact virus has not been identified so far, suggesting that the integration of TEPRVs was not a recent event. The finding of such endogenous virus sequences raises questions concerning 1) the integration process giving rise to endogenous virus sequences, 2) possible differences in the evolutionary rate between the virus and endogenous virus and 3) resistance potential as a result of endogenous virus integration.

In the rice genome, pararetrovirus-like sequences that are similar to rice tungro bacilliform virus (RTBV) have also been found [12-14]. In South and Southeast Asia, rice tungro bacilliform virus, which is transmitted by green leafhoppers, causes one of the most serious diseases of rice with the assistance of rice tungro spherical virus (RTSV) [15]. Kobayashi and Ikeda [16] reported that African rice species, *Oryza glaberrima *and *O. barthii*, showed much severer systemic necrosis compared to the other rice species present in South and Southeast Asia after inoculation of both RTBV and RTSV.

Here, we have characterized RTBV-like sequences in the Japonica (cv. Nipponbare) genome. These sequences, denoted endogenous RTBV-like sequences (ERTBVs), were highly rearranged and dispersed throughout the rice genome. Sequences of the putative viruses for ERTBV were reconstructed from the dispersed segment in the genome. Copy numbers of ERTBV segments are shown to vary among AA-genome *Oryza *species. Asian species have more ERTBV segments than the species originated from the other regions where RTBV is not distributed. The results obtained advance our understanding of the manner of integration of authentic pararetrovirus into the host genome, evolutionary implication of the integrated virus and possible involvement of endogenous virus segments in the corresponding disease resistance.

## Results

### Identification of RTBV-like sequences in the rice genomes

Previously, we found a repetitive sequence near the rice waxy gene, which is partially homologous to the rice tungro bacilliform virus (RTBV) genome [12,17]. A probe of this segment hybridized to about 30 bands in *Eco*RI-digested fragments from both Japonica and Indica genomic DNAs [12]. In the present study, we collected 29 RTBV-like sequences from the rice genome databases for Japonica (cv. Nipponbare). The segments collected here had ample length for further analyses. The segments of ERTBV were distributed throughout the rice genome. Structural differences among the collected ERTBV segments appeared to be due to rearrangements of the segments including deletions, insertions, inversions and duplications. None of the segments had the same structure as the virus or seemed likely to be active as virus. However, the similarities among the ERTBV segments were scored as more than 80% (described in detail in below), so that each homologous part in the ERTBV segments was easily recognized. Most of the ERTBV segments are flanked by AT-repeated sequences (Table 1), which might be involved in the integration mechanism.

**Table 1:** Summary of the ERTBV structures in the rice genomes^1^.

Chr.^2^	Accession Number	ERTBV position (length)^3^	Cluster^4^	alignment of gene^5^					AT-repeated sequence^6^	
				
				Intergenic region	Region x	ORF y (MP/CP/PR)	ORF y (RT/RH)	ORF z	5'-end	3'-end
	[Japonica]									
1	AP003338	31237-38745 (7509)	C	32277-31237	38711-38217	38259-34814	34534-33094	33127-32278	34 (2)	153 (0)
				38745-38712						
2	AP006160^a^	128189-135399^a^	C	128189-128420^a^	128421-128915^a^	128873-129447^a^	134248-132808^a^	132841-131989^a^	97 (0)^a^	12 (0)^b^
	AP004842^b^	1-7610^b^(11686)		131988-131526^a^	7379-6885^b^	130212-131451^a^				
				135399-134914^a^		134913-134534^a^				
				7610-7380^b^		5595-3136^b^				
						6927-6352^b^				
4	AL606628	47001-48868 (1868)	B	-	-	47001-47054	47334-48768	48756-48868	16 (0)	45 (391)
	AL606618	82165-88112 (5948)	B	-	88112-87631	87673-84239	83959-82532	82544-82165	13 (678)	156 (48)
	AL606592	36282-40413 (4132)	B	39437-40413	-	36282-36894	37174-38614	38602-39436	65 (0)	101 (17)
	AL662971	104278-111739 (7462)	C	105283-104278	111720-111226	111268-107823	107543-106102	106135-105284	65 (727)	-
				111739-111721						
	AL607003	63290-71598 (8309)	A	63406-64469	64470-64882	64846-68248	68532-69972	63290-63405	52 (68)	151 (265)
				70794-71598				69939-70793		
5	AC107085	22244-29756 (7513)	B	22436-22244	28859-28374	28416-24971	24691-23258	23270-22437	25 (0)	55 (64)
				29756-28860						
6	AP002542	33961-41438 (7478)	B	39087-38121	38120-37636	37678-34233	39121-40541	40529-41362	216 (2)	252 (1)
				41363-41438						
	AP006056	28996-33700 (4705)	C	29562-28996	-	33700-32101	31821-30381	30414-29563	93 (10)	337 (87)
	AP004750	97242-104472 (7231)	A	98061-97242	104472-103986	104022-100607	100323-98883	98916-98062	39 (178)	52 (694)
7	AP006163	118513-126010 (7498)	C	118513-118518	118519-119013	118971-122416	122696-124136	124103-124954	82 (502)	224 (129)
				124955-126010						
	AP005719	363-7932 (7570)	C	3278-2211	2210-1716	1758-363	5526-4096	4129-3279	65 (100)	33 (2)
						7932-5806				
	AP004348	17568-24897 (7330)	A	18513-17568	24897-24437	24473-21059	20775-19335	19368-18514	96 (100)	37 (20)
8	AP005164	171654-179236 (7583)	C	173775-174838	174839-175333	175291-178760	171654-172956	172923-173774	52 (284)	274 (11)
							179071-179236			
	AP003883	117668-125143 (7476)	C	117668-117743	117744-118238	118196-121641	121921-123361	123328-124179	54 (612)	41 (16)
				124180-125143						
	AP003914	22973-25201 (2229)	A	-	-	-	23074-24514	24481-25201	373 (14)	1136 (0)
	AP005301	34330-41566 (7237)	A	34330-34741	34742-35228	35192-38611	38895-40314	40281-41135	153 (14)	34 (23)
				41136-41566						
	AP005159	88879-97300 (8422)	B	88879-89828	89829-90315	90273-93716	93996-95435	95423-96256	179 (0)	34 (19)
				96257-97300						
9	AP005424	11998-18576 (6579)	C	18576-17867	11998-12492	12450-15849	16129-17569	17536-17833	57 (10)	95 (225)
	AP005860	50709-58281 (7573)	A	50709-50806	50807-51295	51259-54579	54863-56304	56271-57124	218 (0)	8 (3)
				57125-58281						
10	AC119147	89355-102382 (13028)	B	90235-89355	101319-100834	99260-96143	95863-94424	94436-93603	71 (0)	183 (68)
				93602-93432		100876-100548				
				102382-101320						
	AC027660	24299-31769 (7471)	A	29370-28257	28256-27768	27804-24390	31632-30192	30225-29371	-	28 (520)
	AC069300	89901-96619 (6719)	A	90209-89901	96619-96133	96169-92755	92471-91031	91064-90210	21 (162)	21 (688)
11	AC135957	69527-81251 (11725)	A	73392-74286	81251-80765	69527-70846	71130-72570	72537-73391	368 (619)	-
				75398-74475		80801-77387		75444-75410		
12	AL713945	45991-49426 (3436)	B	47029-45991	-	-	49291-47851	47863-47030	55 (1)	261 (9)
	AL731743	14231-18608 (4378)	B	17596-18608	14768-14283	14325-14231	15333-16774	16762-17595	-	44 (40)
						14791-15053				
	AL928780	103187-110757 (7571)	A	103187-103406	103407-103893	103857-107273	107665-109105	109072-109926	44 (25)	25 (32)
				109927-110757						
	AL928749	6275-11538 (5264)	A	7149-6275	-	11538-9695	9411-7971	8004-7150	479 (127)	624 (0)
	[Indica]									
4	AB124591	554-4677(4124)	B	3709-4677	-	554-1166	1446-2886	2874-3708	43(0)	105(26)
ND	AB124592	8867-14510(5644)	C	10990-12051	12052-12546	12504-14510	8867-10171	10138-10989	64(273)	unknown
ND	AB124593	158-5914(5757)	C	4214-5914	-	158-1677	1957-3397	3364-4213	157(0)	62(2)

1 The sequences were mined from Japonica database or investigated from phage clones isolated from Indica (IR36) genomic library.

2 Chromosome no. on which the ERTBV segment resides.

3 Nucleotide positions of ERTBV in the registered genomic clone. Parenthesis indicates a length of ERTBV sequence.

4 Clusters into which the individual ERTBV sequences were phylogenetically grouped.

5 The region belongs to the part of ERTBV, and their nucleotide positions in the clone registered in the database.

6 The numbers indicate repetition times of AT at both the ends of ERTBV, and parenthesis indicates distance (bp) from the ERTBV.

a and b These clones contain the identical ERTBV, but both the clones ended within the ERTBV sequence.

### Assembling ERTBV segments

**Figure 1:**
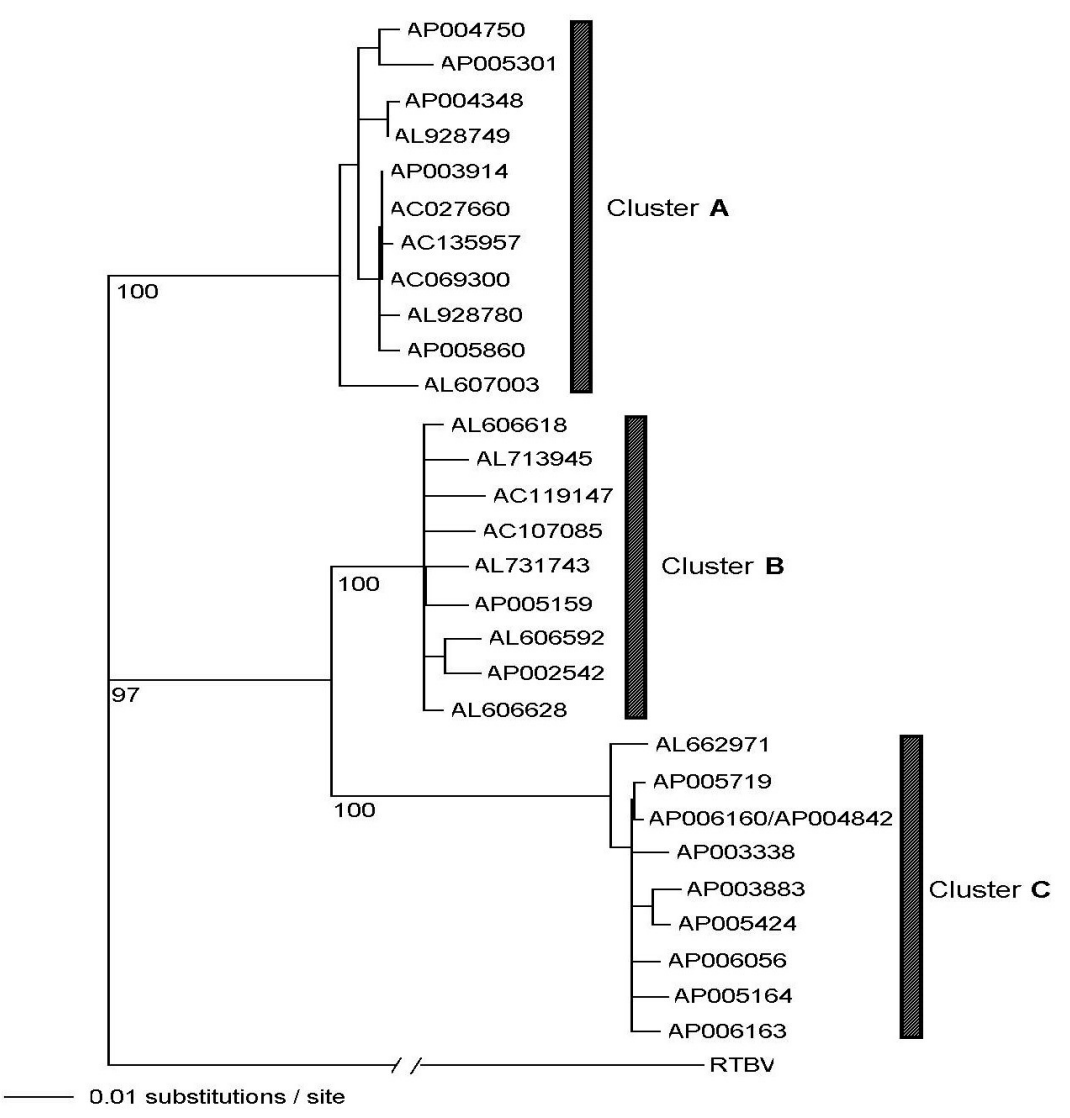
The phylogenetic tree based on reverse transcriptase (RT) gene of 29 ERTBV segments and RTBV. These ERTBV were collected from the rice genome database (Table 1). The nucleotide sequences were aligned using the CLUSTAL W program [35] from the DNA Data Bank Japan (DDBJ). The method detailed of construction of the tree was described in the text. These sequences were fallen into three clusters, ERTBV-A, -B and -C. Numbers above the nodes are bootstrap support based on 100 bootstrap replicates for all branches were resolved on the strict consensus tree.

**Figure 2:**
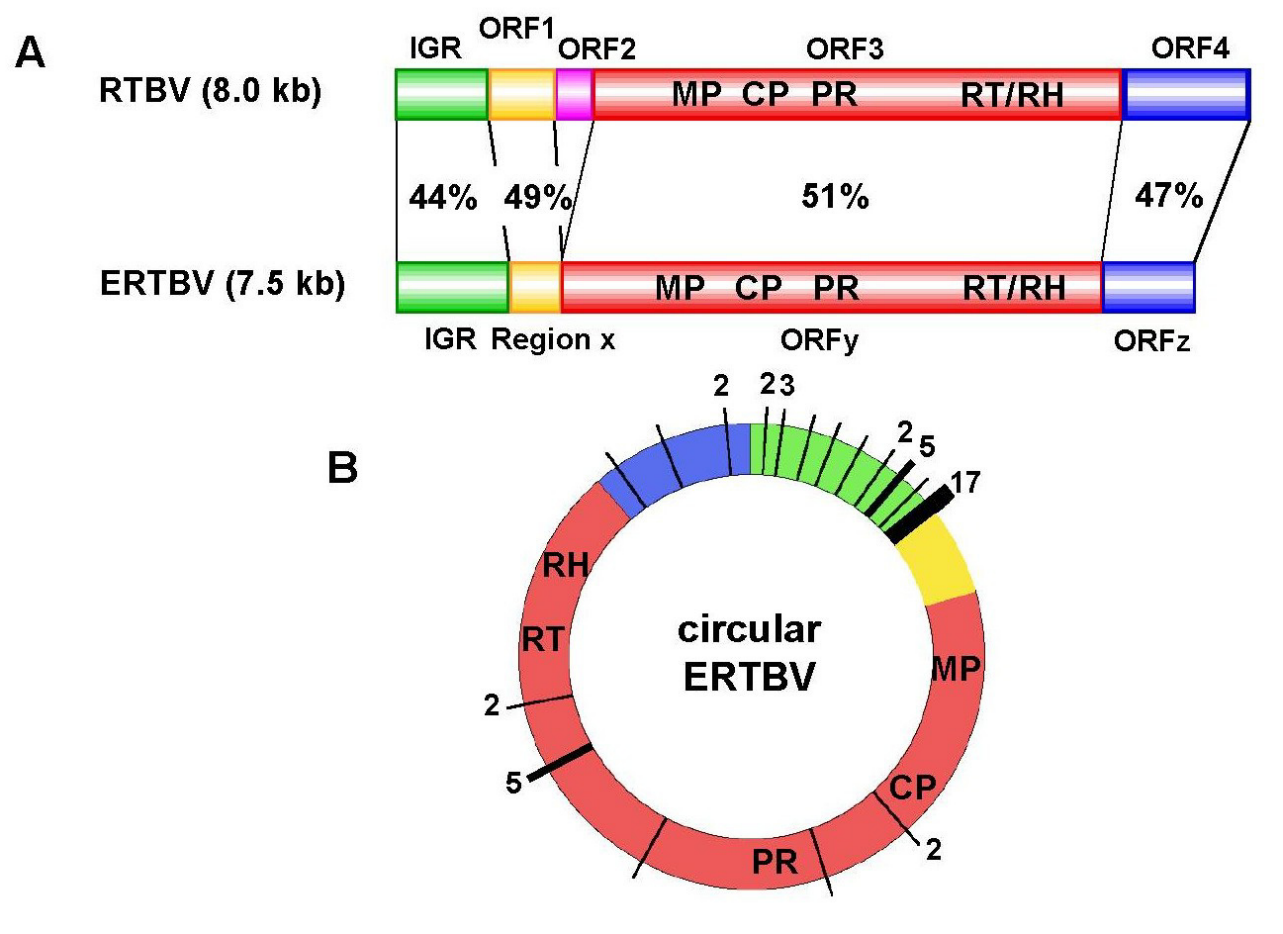
Deduced virus form of ERTBV that was assembled from the rice genomic sequences. A: Comparison of the assembled ERTBV and RTBV. Percentages indicate the nucleotide similarity of each of the corresponding segments or ORFs between ERTBV and RTBV. ERTBV lacks ORF 1 and 2. The assembled sequences designated as ERTBV-A, -B and -C have 7526 bp, 7496 bp and 7499 bp in length, respectively. Their assembled sequences consist of intergenic region (IGR) (A: 1–1114; 1114 bp, B: 1–1066; 1066 bp: C: 1–1063; 1063 bp), Region x (A: 1115–1600; 486 bp, B: 1067–1552; 486 bp, C: 1064–1558; 495 bp), ORF y (A: 1570–6702; 5133 bp, B: 1519–6672; 5154 bp; C: 1525–6678; 5154 bp) and ORF z (A: 6702–7523; 822 bp, B: 6672–7493; 822 bp, C: 6678–7496; 819 bp). Identical organizations of ORFs and their orders were observed in their structures. The nucleotide sequence for Region x corresponds with ORF 1, but ATGs for initiation codon were not present. ORF 3 contains movement protein (MP), coat protein (CP), asparatic protease (PR) and RNase H (RT/RH) [19]. B: Schematic representation of the junction sites of the ERTBV segments adjoining the rice genomic sequence. The junctions are indicated by vertical bars on the circular virus form of ERTBV. The figure shows the number of junctions of all the examined segments referred to Table 1. The different colors in the circle correspond to the above-mentioned segments. The junctions are concentrated in the IGR, which contains the transcriptional initiation and terminal overhanging segments.

We sorted out the 29 ERTBV segments using the sequences homologous to the RT gene, which is encoded in the end of ORF 3. Similarity analyses based on the RT gene grouped them into three clusters (Figure 1 and Table 1), suggesting the presence of three independent ERTBV sequence families. The discontinuity of the three clusters allows us to predict independent integration events for each of the ERTBVs into certain rice species genome(s). We then attempted reconstruction of the complete ERTBVs from the three clusters present in the phylogenetic tree. Approximately 7.5-kb circular virus-like structures could be reconstituted by assembling common parts in individual ERTBVs. The assembled virus-like sequences, which are designated as ERTBV-A, -B and -C, encode potentially functional ORFs. The nucleotide similarities among ERTBVs range from 82% to 93%, and therefore the segments are clearly distinguished in any of the clusters. Of the four ORFs in RTBV, ORF 3 and 4 correspond to ORF y and z of ERTBVs, but ORF 2 is absent from ERTBVs (Figure 2A). Nucleotide sequence of ORF 1 showed a 49% of homology to Region x of ERTBVs, while we failed to find ORF from the Region x sequences (Figure 2A). Pararetroviruses form a circular double-stranded DNA genome (Figure 2B), therefore, it is reasonable to believe that the authentic ERTBV viruses had a similar structure. It seems that the integration of ERTBVs did not occur randomly since more than a half of the ERTBV ends connected with rice genomic DNA were in the putative intergenic region (IGR) (Figure 2B). In addition, the other common junctions are found in the middle of ORF y (Figure. 2B), which corresponds to the location of the discontinuities in the open circular form of RTBV [18]. These structures may indicate that the integration process occurred after the reverse transcription of the virus genome.

**Figure 3:**
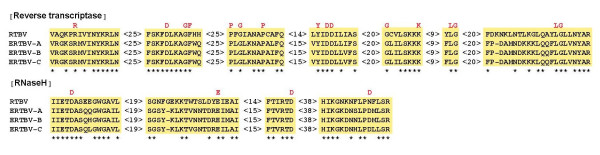
Amino acids sequence alignments for RT and RH domains of RTBV and the assembled ERTBVs. The alignments were performed with the CLUSTAL W program and the conserved motifs are shown encompassed by yellow. The numbers after each motif indicate the numbers of the amino acids, which are not conserved. Conserved amino acid residues are marked with an asterisk. Invariant amino acids are highlighted in red above the sequences.

The longest ORF in RTBV, ORF 3 encodes the movement protein (MP), coat protein (CP), asparatic protease (PR), RT and RNase H (RH) in the single polycistronic mRNA [19]. ORF 3 mostly parallels ORF y in ERTBV-A, -B and -C. Similarities within these nucleotide sequences between the RTBV and ERTBVs were around 50%. The amino acid identity of the genes in ORF 3 ranged from 63% for RT/RH to 40% for PR genes. With respect to RT and RH genes in ORF 3, all characteristic motifs and invariant amino acids for individual genes were preserved in the corresponding ORF in each consensus ERTBV (Figure 3). Therefore, the putative genes in consensus ERTBVs potentially encode proteins comparable to those in RTBV.

**Figure 4:**
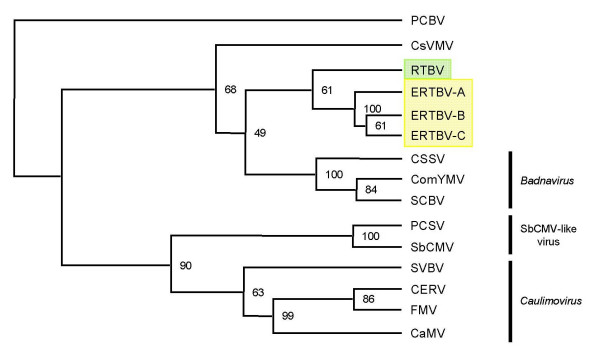
Neighbor-joining dendrogram of putative RT amino acid sequence relationships among species of the different genera of the Caulimoviridae family. The dendrogram was bootstrapped 100 times (percent scores shown at nodes) and rooted on a random sequence.

To evaluate the genetic relationship of ERTBVs and RTBV, RT amino acid sequences from the viruses belonging to Caulimoviridae, were compared. Using the PHYLIP package program [20], the phylogenetic tree was constructed for 14 kinds of viruses in Caulimoviridae (Figure 4). RTBV and ERTBV-A -B and -C were found to be most closely located in the RT peptides dendrogram among the Caulimoviridae viruses. These results strongly suggest that ERTBVs are virus in origin and are closely related with RTBV.

No sequences exactly matching RTBV per se were found in the databases for Japonica cv. Nipponbare. We conducted Southern hybridization analysis to see whether RTBV-homologous sequences are present in 14 lines from *Oryza *AA-genome species (Table 1). The hybridization patterns probed with RTBV sequence resulted in faint and indistinct bands (data not shown).

### Periods of integration of ERTBV segments into the rice genome

To investigate the periods of integration of ERTBV, we attempted to obtain ERTBV sequences from an Indica variety (cv. IR36) using the RTBV-like sequence near the *waxy *locus as a probe. We thereby isolated three clones carrying ERTBV-homologous sequences (Table 1). Indica clone, AB124591, was found to have the same ERTBV sequence as that found in Nipponbare accession AL606592 (Table 1), indicating that the ERTBV integration events occurred before the Japonica-Indica differentiation. The other two Indica clones do not correspond with any ERTBV segments from Nipponbare.

Except for the sequences examined here, we could not find any other RTBV-like sequences in database searches using RTBV nor each of ERTBVs sequences as queries. Therefore, a fourth cluster of the RTBV-like sequences is unlikely to be present. The integration of ERTBV or its derived segments after the differentiation of Japonica and Indica cultivars thus seems to have not occurred.

### Distribution of ERTBVs in the *Oryza *AA-genome species

**Figure 5:**
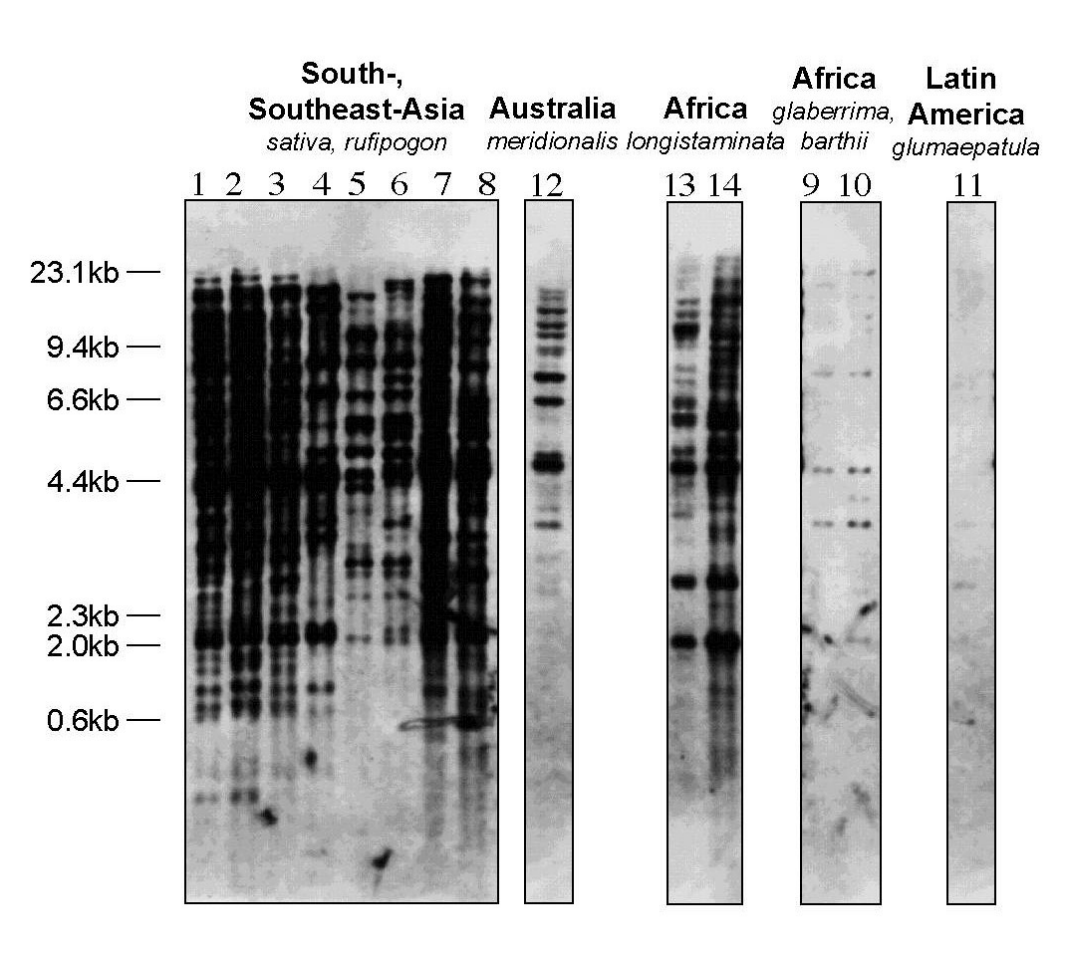
Southern blotting patterns for the genomic DNAs from 14 *Oryza *lines probed with 7.4-kb ERTBV fragment. The numbers above the blots correspond to the numbers of the materials (Table 2). The regions are the origins of the materials. Some accessions/lines of *O. sativa*, *O. rufipogon *and *O. longistaminata *are being used as the sources of resistance against tungro disease..

To test whether other *Oryza *species contain ERTBV in their genomes, Southern blotting experiments were performed with 14 lines of the *Oryza *AA-genome species. A PCR fragment of the 7.4-kb ERTBV sequence from chromosome 10 was prepared as a probe, and Figure 5 shows the discrete bands and varying copy numbers revealed by the hybridization patterns. Each accession or cultivar of *O. sativa *and *O. rufipogon *(lanes 1–8) has about 50 bands, some of which showed common sizes among the lines. An Australian rice (*O. meridionalis*) and two *O. longistaminata *accessions showed middle-copy numbers (approximately 10–20) of the ERTBV segments. In *O. glaberrima*, *O. barthii *and *O. glumaepatula *accessions, only a few bands hybridized to ERTBV; this may also be caused by divergence of the ERTBV sequence and not only due to lower copy numbers of the sequence. Especially in These three species derived from Africa and Latin America were genetically distinct from Asian rice species, but another African rice species,*O. longistaminata, *was considered to have undergone introgression with Asian species [21]. Africa and Latin America are regionally isolated from rice tungro disease.

### Methylation of ERTBV in *Oryza *AA-genome species

**Figure 6:**
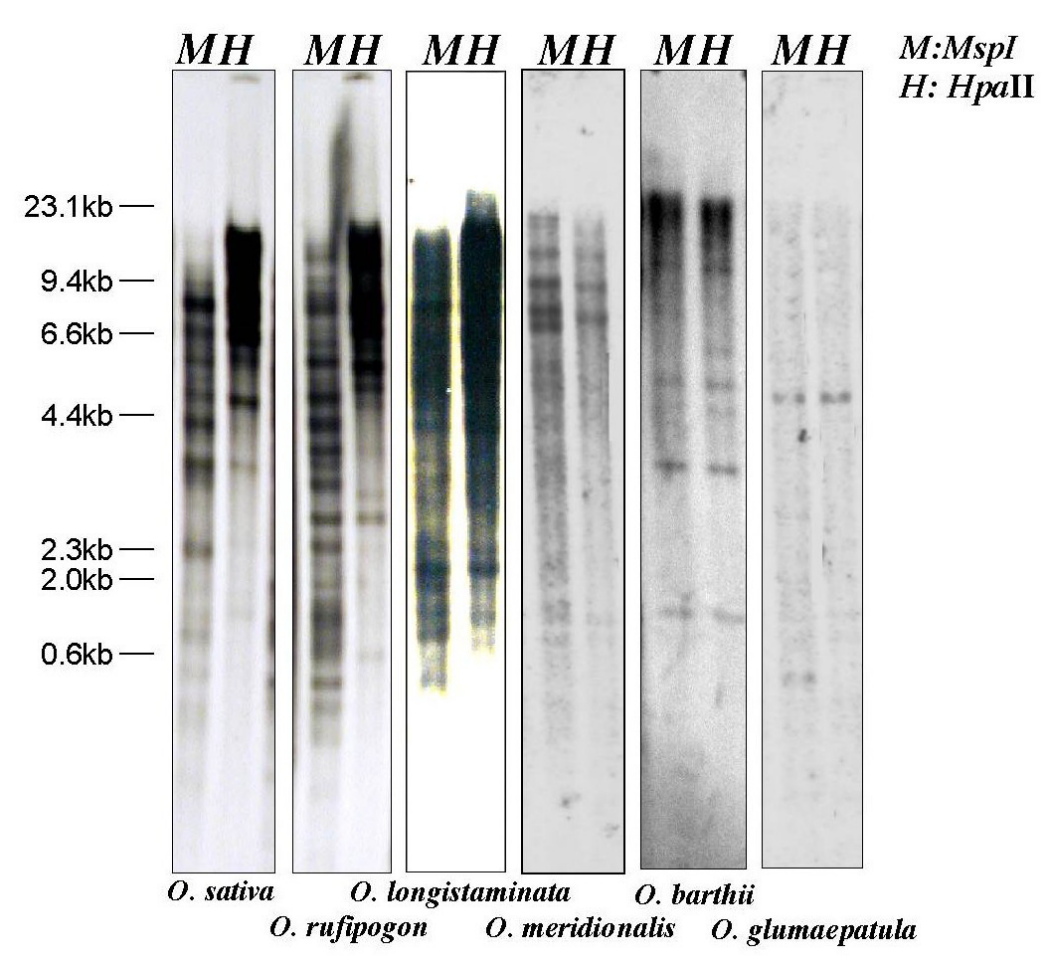
Methylation state of ERTBV in the genomic DNAs from the *Oryza *AA-genome species. Each genomic DNA was digested with *Msp*I (M) or *Hpa*II (H) as described in Materials and Methods. Differential hybridization patterns between the *Msp*I and *Hpa*II digests observed in *O. sativa *and *O. rufipogon *show heavier methylation compared to the others, and *O. longistaminata *indicates possession of methylated ERTBV segments in the genome.

Since high-copy-number sequences generally tend to have a heavier methylation state than low-copy-number sequences, we investigated the methylation state of ERTBVs in several *Oryza *species with various copy numbers. The methylation states were analyzed by Southern blotting method using a methylation-sensitive enzyme, *Hpa*II, and a partially methylation-sensitive isoschizomer, *Msp*I. The 7.4-kb probe was employed to examine the methylation states of the ERTBV in the *Oryza *AA-genome species. The blotting patterns of *O. sativa *(Shimokita), *O. rufipogon *(W1954), *O. longistaminata *(W1034), *O. meridionalis *(W1625), *O. barthii *(W1592), *O. glumaepatula *(W1185) showed differences of methylation state depending on the ERTBV copy number (Figure 6). In the high-copy-number species, *O. sativa *and *O. rufipogon*, the *Msp*I and *Hpa*II digests of DNA clearly showed different digestion patterns, and in particular, most of the small bands in the *Msp*I digests were not observed in the *Hpa*II digests, indicating that these ERTBV sequences were considerably methylated. Although the results of blotting for the middle-copy-number species, *O. longistaminata *and *O. meridionalis*, also showed the presence of methylcytosine within their ERTBV sequences, some bands in the *Msp*I digests were shared with the *Hpa*II digests. Preferential digestion of DNA from the low-copy-number species, *O. barthii *and *O. glumaepatula*, compared to DNA from the other species was observed. The above results indicate that the copy number of the ERTBV sequences in the *Oryza *genomes is correlated with the methylation level. A chloroplast DNA fragment [22] was used as a control probe to confirm the completeness of digestions (data not shown).

## Discussion

### Completion of ERTBV via integration of the virus

**Figure 7:**
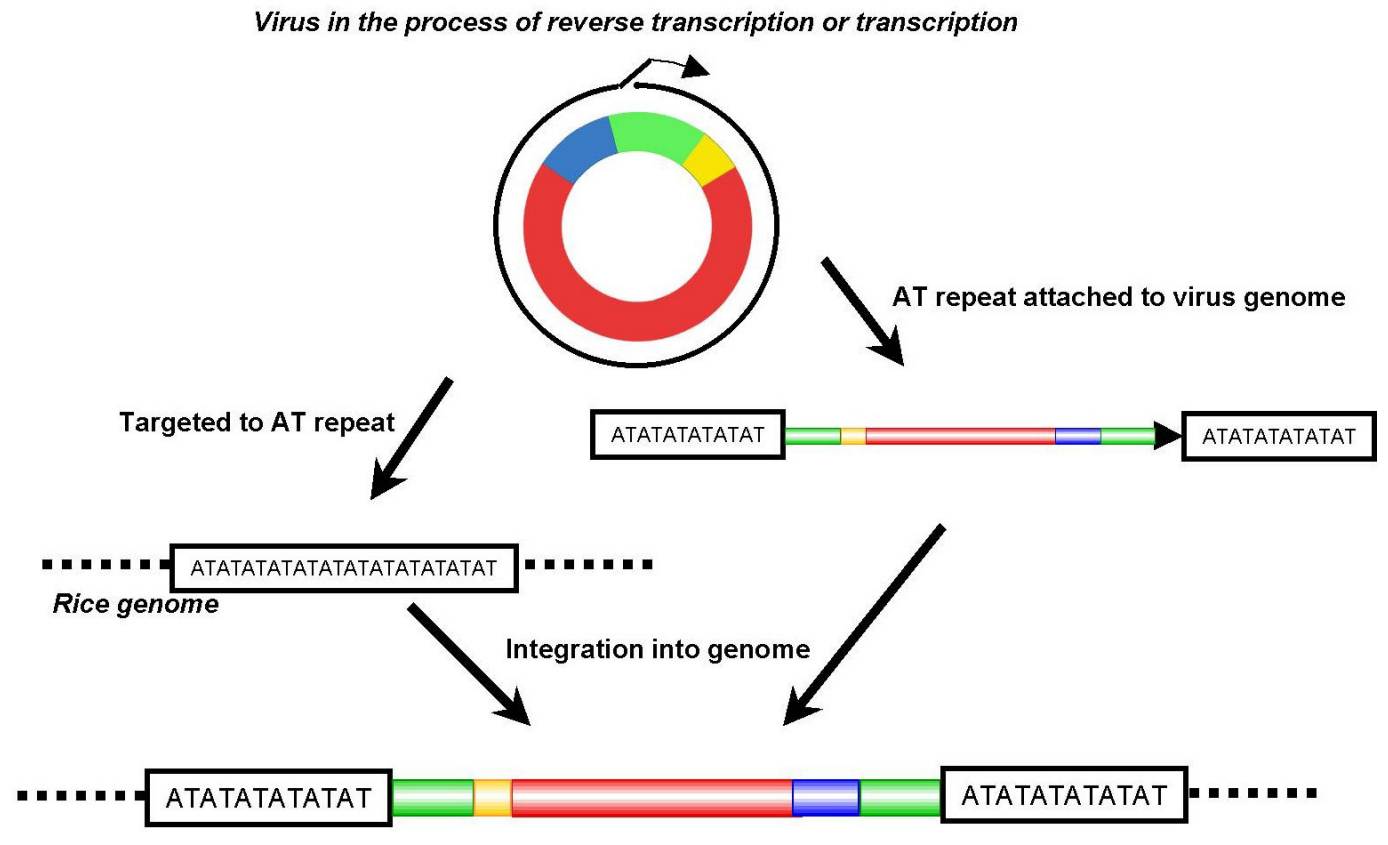
Two hypothetical processes for the integration of the putative virus for ERTBV into the rice genome. DNA strands after the reverse transcription step of the virus were targeted to AT-repeat sequences (left), or AT-repeat sequences became attached to the virus segments during the process of the reverse transcription and the complex was integrated into the rice genome (right).

Our data suggest that the putative virus for ERTBVs has been integrated into the *Oryza *genomes at least three times. These putative viruses are thought to be closely related to RTBV and to form circular double-stranded DNA. Cleavage of circular DNA molecules or open circular forms should be required for integration of the infecting virus. One of the preferential cleavage sites of ERTBV was mapped within a putative promoter segment of the authentic ERTBV sequence. Integrated sequences of TPVL in tobacco [8] and hepatitis B (hepadna) virus in human liver [23] were also found to have preferred junctions with their host genomes at a similar region to that seen in ERTBV. Jakowitsch *et al*. [8] proposed that the open circular form of the virus during the process of replication was involved in integration, and the free ends of the virus DNA molecule might contribute to integration or recombination. The ends of the ERTBV segments likely correspond with IGR and the putative discontinuities of RTBV (Figure 2B), which possibly functions in transcription and replication initiation or in the priming of DNA strand synthesis [18]. This fact accords with Jakowitschs' idea that the preferential integration occurred while the virus was in the process of replication (Figure 7). In addition to the preferred sites for integration within the virus DNA, we found that 93% of the ERTBV ends were flanked by AT-repeated sequences. This high probability of the presence of AT-repeated sequences adjoining ERTBV led us to postulate that the AT tracks have facilitated or are associated with integration of the virus DNA into the host genome (Figure 7). Transgenes introduced by particle bombardment into the *Arabidopsis *genome were preferentially delivered to AT-rich scaffold/matrix-attached region (S/MAR)-like sequences [24,25]. SINE integration sites in the *Brassica *genome show strong affinity for S/MAR-like sequences [26]. Similar processes accounting for these two instances might also function in the case of integration of the virus for ERTBV. Our data alone, however, are unable to distinguish between whether ERTBV integrated into the AT-repeat sequences or whether ERTBV accompanied the AT-repeat sequences in their integration (Figure 7).

### Relationship between ERTBV and RTBV

Although the genomes of ERTBV and RTBV are structurally similar, particularly in the longest ORF, some regions are markedly different. Phylogenetically, both are apparently closely related viruses. One important question is whether or not ERTBV is a cognate of RTBV. ERTBVs existed in the *Oryza *AA-genome species before differentiation of Japonica and Indica. Considering this evolutionary period, two possible relationships of RTBV and ERTBV might be predicted: one is that the virus that was to become ERTBV was a direct progenitor of RTBV, and the other is that both were differentially branched from a common ancestral virus. Because the evolution of virus genomes is generally much faster than that of plant genomes, we cannot compare them equivalently. In fact, rapid evolution of RTBV was inferred from high level of genetic diversity of RTBV field isolates [27]. Even in a single field in the Philippines, more than one RTBV isolate could be observed, and the genotype changes year by year. Comparison of the sequences of several different isolates revealed evidence for incidences of nucleotide substitution, insertion/deletion and recombination occurred during differentiation of RTBV isolates [28]. Particularly, it was suggested that recombination had played a role in the evolution of RTBV [29]. If ERTBV is a direct progenitor of RTBV, recombination events might have contributed as a driving force to establish the present RTBV form. The longest ORF (ORF 3) of RTBV is functionally essential [30] and is thought to have been conserved since ERTBVs were present as viruses, but the other less homologous segments might have gradually undergone substitution by recombination. If RTBV was derived from the virus that became ERTBV, estimation of the nucleotide substitution rate of the genes common to RTBV and ERTBV would allow us to compare the evolutionary rates of a plant genome and a plant virus. By such a comparison, we estimated the virus evolution ranged from 30 to140 times faster than that of the host genome. The faster evolution of a virus was thus substantiated for the first time in plants if the virus for ERTBV was in fact the progenitor of RTBV. The different evolutionary rates are dependent on the virus genes; that is, the slow evolution might reflect the functional conservation of the gene.

In the other case, in which RTBV and the virus for ERTBV are phylogenetically located on different branches, the progeny of the virus corresponding to ERTBV might have vanished or may be hidden in a small population. Even though RTBV is not in the case a direct progeny of the virus corresponding to ERTBV, their structural similarity and parallel distribution lead us to consider their common ancestral origin.

### Correlation between rice tungro disease and ERTBV

Plant virus-like sequences have been found in several plant genomes such as banana, tobacco and petunia [11,31]. So far, no association between virus disease and endogenous virus sequences has been reported [32]. Kobayashi and Ikeda [16] reported that *O. glaberrima *and *O. barthii *showed severe systemic necrosis within 4 weeks after infection of RTBV and rice tungro spherical virus (RTSV). *O. longistaminata*, which possesses ERTBV fragments in its genome, showed disease symptoms like those of the Asian species, and some of the accessions were utilized for obtaining the resistance gene [29]. Interestingly, this species originated from Africa where RTBV is not distributed. The fact led us to suppose that there is a correlation between presence or absence of ERTBV in the genome and the degree of RTBV susceptibility. The species, which have a low-copy-number of ERTBV tend to be vulnerable to the rice tungro disease caused by both RTBV and RTSV. The methylation of ERTBV appeared to be positively related in the copy number in the genome. Based on a study on the endogenous virus sequences (EPRV) in tobacco, Mette *et al*. [33] proposed a model in which methylation dependent on the copy number of endogenous virus sequences may induce episomal viral methylation through a homology-dependent process involving DNA-DNA or RNA-DNA interaction. The phenomenon observed here fits their model. The copy number of EPRV in the tobacco genome is 10 times as high as that of ERTBV in the rice genome. Our results demonstrated that about 50 copies of endogenous elements are sufficient to induce methylation in the genome. If we ever find the rice germ lines that have incorporated sequences more similar to those of RTBV, as well as ERTBV in germ lines, those would be exploited as valuable sources of stronger resistance against the rice tungro disease.

## Conclusions

The rice genome contains more than 30 of RTBV-like sequence (ERTBVs) which were unlikely to have functional potential as a virus, while we were able to assemble putative virus forms from these sequences. The phylogenetic analysis showed that at least three times integrations of authentic ERTBVs occurred during *Oryza *speciation. ERTBV integrations likely occurred when the virus was in the replication process, and were preferentially targeted to AT-repeat sequences. The closely relationship between ERTBV and RTBV were proven by comparisons of the DNA and amino acid sequences. The *Oryza *AA-genome species originated from RTBV-distributed regions appeared to contain higher copy numbers of ERTBV segments. The methylation state of the ERTBV sequences was correlated with their copy number in the genome. The results obtained allowed us to speculate a possible relationship between RTBV disease resistance and the copy number and/or DNA methylation of ERTBV in the *Oryza *AA-genome species.

## Methods

### ERTBV sequences from Japonica

Sequences of ERTBV in Japonica (cv. Nipponbare) were mined with rice blast search queries [34] against the rice genome sequences that had been registered as of June 2003. Twenty-nine ERTBV sequences were found in the following Japonica genomic database (Table 1).

### ERTBV sequences from Indica

We attempted to search for ERTBV sequences in the Indica (cv. 93–11) genome database, however, the homologous sequences found through the search had insufficient length for designation as ERTBV segments. Indica ERTBV sequences were isolated from the EMBL 3 genomic library constructed with IR36 strain (FL1041j), which was purchased from Clontech (Palo Alto, California). For screening, a 3.5-kb ERTBV fragment about 50-kb upstream of the *waxy *locus was used as probe [17]. Three clones carrying an ERTBV-containing segment of more than 4 kb were selected. Nucleotide analysis was performed with using a d-Rhodamine Terminator Cycle Sequencing Ready Reaction-Sequencing Kit (Applied Biosystems) and an ABI377 Automated DNA Sequencer (Applied Biosystems).

### Construction of phylogenetic trees

The nucleotide sequences and amino acids sequences inferred from the reverse transcriptase (RT) gene were aligned using the CLUSTAL W program [35] from the DNA Data Bank Japan (DDBJ). We calculated the pairwise nucleotide divergence (K) between 30 independent ERTBV sequences (including RTBV) based on Kimura's two-parameter method [36] without taking synonymous and nonsynonymous changes into account. We constructed a neighbor-joining (NJ) tree based on these estimates [37]. The tree was drawn using PAUP*4.0 [38]. The consensus maximum parsimony and NJ trees based on amino acid sequences for RT in 14 viruses including ERTBV-A, -B and -C were calculated using programs from the PHYLIP package [20]. The minimum evolution tree was calculated by the implementation of PAUP software in the GCG package.

### Southern hybridization

Total genomic DNA was isolated from 7 *Oryza *AA-genome species including 14 strains (Table 2). *Eco*RI-digested DNA was separated by 0.7% agarose gel electrophoresis. The resultant DNA was transferred to nylon membranes (Pall: Biodyne B), and hybridized using the Alk Phos Direct Southern hybridization kit (Amersham Life Science). For the probe, nearly the full length of the 7.4-kb ERTBV fragment (located on chromosome 10, BAC clone accession no. AC069300) was amplified by PCR with the primer combination of 5'GAACTACAACTAGATATGAACGGGGATA3'+5'CACAACTATTCTTAGTGCTGAATTCACTT3'. The membranes were washed twice under the standard Alk Phos conditions with 0.5 M NaCl at 42°C for 20 minutes. To test the methylation state of ERTBV, Southern blotting analysis was carried out using the C-methylation-sensitive enzyme *Hpa*II and the partially sensitive enzyme *Msp*I (isoschizomer of *Hpa*II). The same probe mentioned above was prepared using a PCR-based labeling system with the PCR DIG labeling mix (Roche). To verify that complete digestion was achieved by the enzymes, a chloroplast DNA fragment, the 5.2-kb Sma-8 fragment of buckwheat [22], was used as a control probe.

**Table 2:** Plant materials used.

Sample	*Species*	Cultivar or accession	Remarks
1	*O. sativa*	Shimokita	Japonica from Japan
2	*O. sativa*	T65wx	Near-isogenic line of Taichung 65 with wx from Kinoshitamochi (BC12)
3	*O. sativa*	221	Javanica type from Indonesia
4	*O. sativa*	PTB10	Indica type from India
5	*O. rufipogon*	W107	Annual type from India
6	*O. rufipogon*	W120	Perennial type from India
7	*O. rufipogon*	W1717	Perennial type from China (through IRRI)
8	*O. rufipogon*	W1718	Perennial type from China (through IRRI)
9	*O. glaberrima*	W025	From Guinea
10	*O. barthii*	W1592	From Cameroon
11	*O. glumaepatula*	W1185	From Surinam
12	*O. meridionalis*	W1625	From Australia
13	*O. longistaminata*	W1034	From Nigeria
14	*O. longistaminata*	W1572	From Nigeria

### Sequence data

The sequences containing Indica ERTBV have been deposited in DDBJ: accession nos. AB124591, AB124592 and AB124593. The reconstructed sequences for the authentic ERTBV viruses, ERTBV-A, -B and -C were deposited in DDBJ: accession nos. BR000029, BR000030 and BR000031, respectively.

## Authors' contributions

MoKu and MaKa carried out the molecular genetic studies and participated in the sequence alignments. HN participated in the sequence alignment and performed the phylogenetic analysis. IU participated in the design of the study. YK conceived of the study and drafted the manuscript. YK and YS participated in its design and coordination. All authors read and approved the final manuscript.

## References

[B1] Bejarano ER, Khashoggi A, Witty M, Lichtenstein C (1996). Integration of multiple repeats of geminiviral DNA into the nuclear genome of tobacco during evolution. Proc Natl Acad Sci U S A.

[B2] Ashby MK, Warry A, Bejarano ER, Khashoggi A, Burrell M, Lichtenstein CP (1997). Analysis of multiple copies of geminiviral DNA in the genome of four closely related Nicotiana species suggest a unique integration event. Plant Molecular Biology.

[B3] Richert-Poggeler KR, Shepherd RJ (1997). Petunia vein-clearing virus: A plant pararetrovirus with the core sequences for an integrase function. Virology.

[B4] Richert-Poggeler KR, Noreen F, Schwarzacher T, Harper G, Hohn T (2003). Induction of infectious petunia vein clearing (pararetro) virus from endogenous provirus in petunia. Embo Journal.

[B5] Harper G, Hull R (1998). Cloning and sequence analysis of banana streak virus DNA. Virus Genes.

[B6] Ndowora T, Dahal G, LaFleur D, Harper G, Hull R, Olszewski NE, Lockhart B (1999). Evidence that badnavirus infection in Musa can originate from integrated pararetroviral sequences. Virology.

[B7] Harper G, Osuji JO, Heslop-Harrison JS, Hull R (1999). Integration of banana streak badnavirus into the Musa genome: Molecular and cytogenetic evidence. Virology.

[B8] Jakowitsch J, Mette MF, van der Winden J, Matzke MA, Matzke AJM (1999). Integrated pararetroviral sequences define a unique class of dispersed repetitive DNA in plants. P Natl Acad Sci U S A.

[B9] Lockhart BE, Menke J, Dahal G, Olszewski NE (2000). Characterization and genomic analysis of tobacco vein clearing virus, a plant pararetrovirus that is transmitted vertically and related to sequences integrated in the host genome. J Gen Virol.

[B10] Gregor W, Mette MF, Staginnus C, Matzke MA, Matzke AJ (2004). A distinct endogenous pararetrovirus family in Nicotiana tomentosiformis, a diploid progenitor of polyploid tobacco. Plant Physiol.

[B11] Harper G, Hull R, Lockhart B, Olszewski N (2002). Viral sequences integrated into plant genomes. Annual Review of Phytopathology.

[B12] Nagano H, Kawasaki S, Kishima Y, Sano Y (2000). Structural differences in the vicinity of the waxy locus among the Oryza species with the AA-genome: identification of variable regions. Theoretical and Applied Genetics.

[B13] Mao L, Wood TC, Yu YS, Budiman MA, Tomkins J, Woo SS, Sasinowski M, Presting G, Frisch D, Goff S, Dean RA, Wing RA (2000). Rice transposable elements: A survey of 73,000 sequence-tagged-connectors. Genome Research.

[B14] Nagano H, Kunii M, Azuma T, Kishima Y, Sano Y (2002). Characterization of the repetitive sequences in a 200-kb region around the rice waxy locus: diversity of transposable elements and presence of veiled repetitive sequences. Genes & Genetic Systems.

[B15] Hibino H,, Saleh N,, Roechan M, (1979). Transmission of two kinds of rice tungro-associated viruses by insect vectors. Phytopathology.

[B16] Kobayashi N,, Ikeda R, (1992). Necrosis caused by rice tungro viruses in Oryza glaberrima and O. barthii. Japanese Journal of Breeding.

[B17] Nagano H,, Oka A,, Kishima Y,, Sano Y, (2000). DNA sequences homologous to rice tungro bacilliform virus (RTBV) present in the rice genome.. Rice Genetic Newsletter.

[B18] Hull R (1996). Molecular biology of rice tungro viruses. Annual Review of Phytopathology.

[B19] Herzog E, Guerra-Peraza O, Hohn T (2000). The Rice tungro bacilliform virus gene II product interacts with the coat protein domain of the viral gene III polyprotein. J Virol.

[B20] Felsenstein J,, University of Washington (1995). Phylip Phylogenetic Inference Package.

[B21] Chu YE,, Oka HI, (1970). Introgression across isolating barriers in wild and cultivated Oryza species. Evolution.

[B22] Kishima Y, Ogura K, Mizukami K, Mikami T, Adachi T (1995). Chloroplast DNA Analysis in Buckwheat Species - Phylogenetic-Relationships, Origin of the Reproductive Systems and Extended Inverted Repeats. Plant Science.

[B23] Sherker AH, Marion PL (1991). Hepadnaviruses and Hepatocellular-Carcinoma. Annual Review of Microbiology.

[B24] Sawasaki T, Takahashi M, Goshima N, Morikawa H (1998). Structures of transgene loci in transgenic Arabidopsis plants obtained by particle bombardment: Junction regions can bind to nuclear matrices. Gene.

[B25] Liebich I, Bode J, Reuter I, Wingender E (2002). Evaluation of sequence motifs found in scaffold/matrix-attached regions ( S/MARs). Nucleic Acids Research.

[B26] Tikhonov AP, Lavie L, Tatout C, Bennetzen JL, Avramova Z, Deragon JM (2001). Target sites for SINE integration in Brassica genomes display nuclear matrix binding activity. Chromosome Research.

[B27] Arboleda M,, Azzam O, (2000). Inter- and intra-site genetic diversity of natural field populations of rice tungro bacilliform virus in the Philippines. Archives of Virology.

[B28] Cabauatan PQ, Melcher U, Ishikawa K, Omura T, Hibino H, Koganezawa H, Azzam O (1999). Sequence changes in six variants of rice tungro bacilliform virus and their phylogenetic relationships. J Gen Virol.

[B29] Cabunagan RC,, Angeles ER,, Villareal S,, co-authors 16, Chancellor T C B, Azzam O and Heong K L (1999). Multilocation evaluation of advanced breeding lines for resistance to rice tungro viruses. Rice Tungro Disease Management.

[B30] Laco GS, Beachy RN (1994). Rice Tungro Bacilliform Virus Encodes Reverse-Transcriptase, DNA-Polymerase, and Ribonuclease-H Activities. Proc Natl Acad Sci U S A.

[B31] Hull R, Harper G, Lockhart B (2000). Viral sequences integrated into plant genomes. Trends in Plant Science.

[B32] Covey SN, Al-Kaff NS (2000). Plant DNA viruses and gene silencing. Plant Molecular Biology.

[B33] Mette MF, Kanno T, Aufsatz W, Jakowitsch J, van der Winden J, Matzke MA, Matzke AJM (2002). Endogenous viral sequences and their potential contribution to heritable virus resistance in plants. Embo Journal.

[B34] RiceBLAST: Rice sequence database BLAST search. http://riceblast.dna.affrc.go.jp/.

[B35] Thompson JD, Higgins DG, Gibson TJ (1994). Clustal-W - Improving the Sensitivity of Progressive Multiple Sequence Alignment through Sequence Weighting, Position-Specific Gap Penalties and Weight Matrix Choice. Nucleic Acids Research.

[B36] Kimura M, (1980). A simple method for estimating evolutionary rates of base substitutions through comparative studies of nucleotide sequences. Journal of Molecular Evolution.

[B37] Saitou N, Nei M (1987). The Neighbor-Joining Method - a New Method for Reconstructing Phylogenetic Trees. Mol Biol Evol.

[B38] Swofford DL,, Sinauer Association (1998). PAUP*. Phylogenetic Analysis Using Parsimony (*and Other Method).

